# Patient Satisfaction and Perioperative Outcomes of Wide-Awake Local Anesthesia No Tourniquet Versus Supraclavicular Peripheral Nerve Block in Elective Hand and Forearm Surgery: A Prospective Comparative Study

**DOI:** 10.3390/jcm15062360

**Published:** 2026-03-19

**Authors:** Mustafa Azizoğlu, Argun Pire, Levent Özdemir, Aslınur Sagün, Erdi Hüseyin Erdem, Melikşah Soylu, Ender Gümüşoğlu, Emre Öztürk

**Affiliations:** 1 Anesthesia and Reanimation Department, Mersin University, 33110 Mersin, Turkey; argunp@gmail.com (A.P.); leventozdemirmd@gmail.com (L.Ö.); aslinur_aslan@hotmail.com (A.S.); h.erdierdem@gmail.com (E.H.E.); meliksahsoylu@gmail.com (M.S.); 2 Orthpaedics and Traumatology Department, Mersin University, 33110 Mersin, Turkey; endergumusoglu@gmail.com (E.G.); emreozturk@mersin.edu.tr (E.Ö.)

**Keywords:** regional anesthesia, hand injuries, patient outcome assessment, patient satisfaction

## Abstract

**Background/Objectives**: Wide Awake Local Anesthesia No Tourniquet (WALANT) and ultrasound-guided peripheral nerve blocks (PNBs) are increasingly used alternatives to general anesthesia for hand and forearm surgery. While WALANT is commonly perceived as a time-efficient and resource-sparing technique, comparative data regarding patient satisfaction, perioperative pain, and time-related outcomes remain inconsistent. This study aimed to compare WALANT and ultrasound-guided supraclavicular peripheral nerve block techniques with respect to patient satisfaction, perioperative pain, time-related parameters, and surgeon-related outcomes in elective hand and forearm extremity surgery. **Methods**: This prospective comparative observational study included 80 adult patients undergoing elective hand or forearm surgery. Patients received either WALANT or ultrasound-guided supraclavicular brachial plexus block according to patient preference. The primary outcome was overall patient satisfaction assessed within 24 h postoperatively. Secondary outcomes included block performance time, waiting time, total anesthesia-related time, intraoperative and postoperative pain scores, additional sedation requirements, postoperative numbness, willingness to choose the same anesthetic technique again, safety outcomes and surgeon satisfaction. **Results**: Overall patient satisfaction was significantly higher in the peripheral nerve block group compared with the WALANT group (median [IQR]: 90 [85–100] vs. 80 [70–90], *p* < 0.0001). Intraoperative and postoperative pain scores were also significantly lower in the peripheral nerve block group. Although block performance time was longer with the peripheral nerve block, waiting time and total anesthesia-related time were significantly shorter compared with WALANT. Surgeon satisfaction and the need for additional intraoperative sedation did not differ significantly between groups. **Conclusions**: In elective hand and forearm surgery, ultrasound-guided supraclavicular peripheral nerve block was associated with higher patient satisfaction, lower pain scores, and shorter total anesthesia-related time compared with WALANT. Surgical satisfaction scores were similar with both anesthetic techniques. Considering the heterogeneity of clinical settings and procedural requirements, as well as cost and resource utilization considerations, anesthetic technique selection should be individualized.

## 1. Introduction

Hand and forearm surgeries remain clinically important, as they account for approximately one-fifth of all emergency department admissions and represent one of the most frequently encountered case groups in orthopedic surgical practice [1]. Advances in regional anesthesia techniques have contributed to reduced postoperative pain and improved clinical outcomes, while the Wide Awake Local Anesthesia No Tourniquet (WALANT) technique has enabled selected procedures to be performed in resource-limited settings in appropriately selected patients [2].

The increasing use of ultrasonography has improved the safety and success rates of peripheral nerve block (PNB) techniques [3]. In addition to higher success rates, prolonged block duration has been associated with reduced postoperative analgesic requirements [4,5]. In recent years, WALANT has emerged as an alternative anesthetic approach, and its potential advantages over PNBs -including the absence of routine anesthesiologist involvement and relatively shorter application time- have been increasingly discussed [6,7].

With the establishment of the safety of local anesthetic and epinephrine combinations, the WALANT technique has gained growing popularity [8]. This approach eliminates the need for sedation or general anesthesia, thereby reducing the risk of systemic complications such as nausea, vomiting, and respiratory depression, and offering a particularly safe option for high-risk patients. Maintaining patient consciousness during surgery allows active participation through intraoperative movement in procedures such as tendon repair and may reduce postoperative opioid requirements. Furthermore, avoiding tourniquet use decreases the risk of tourniquet-related pain and neuropraxia, enabling surgeons to work without time constraints and allowing effective hemostasis even in patients receiving anticoagulant therapy [9].

Both WALANT and PNB techniques offer tangible benefits compared with general anesthesia in hand surgery, including reduced per-case costs, shorter procedural cycles, and preservation of functional testing [7]. WALANT is commonly regarded as a time-efficient technique that reduces anesthetic workload by avoiding sedation and extensive anesthetic preparation and is typically performed by the surgeon. Both WALANT and regional anesthesia have been associated with high patient satisfaction in hand and forearm surgery; however, satisfaction is influenced by multiple perioperative factors. With the increasing emphasis on patient-centered care, patient satisfaction has become an important outcome alongside traditional analgesic measures [10]. Understanding how different anesthetic strategies influence not only pain control but also overall patient experience is therefore essential for optimizing perioperative care.

This study aimed to comprehensively compare WALANT and PNB techniques across clinically relevant perioperative domains. Patient satisfaction and patients’ willingness to choose the same anesthetic technique again were the primary outcomes assessed within 24 h postoperatively. Block performance time, waiting time, total procedural time, additional sedation requirements, intraoperative and postoperative pain and numbness, surgeon satisfaction, as well as safety outcomes were defined as secondary outcomes.

## 2. Materials and Methods

### 2.1. Study Design

This prospective observational comparative study was conducted after approval was obtained from the Mersin University Clinical Research Ethics Committee (Approval No: 221, Date: 3 March 2021), in accordance with the principles of the Declaration of Helsinki. Patients scheduled for elective hand and forearm surgery at our institution between January 2024 and December 2024 were screened for eligibility. Written informed consent was obtained from all participants after detailed preoperative information was provided. This study was conducted and reported in accordance with the STROBE guidelines.

### 2.2. Study Population and Patient Selection

The study population consisted of patients aged 18–65 years with an American Society of Anesthesiologists (ASA) physical status classification of I–III who were scheduled to undergo forearm or hand surgery (including carpal tunnel release and nerve/artery/tendon repair). Exclusion criteria included known allergy to local anesthetics, infection at the injection site, severe coagulopathy, severe psychiatric disorders, opioid dependence, procedures expected to last longer than 60 min, conversion to general anesthesia and refusal of regional anesthesia.

A total of 80 patients meeting the inclusion criteria were enrolled during the study period. Patients were first asked whether they would accept undergoing the procedure under an awake anesthetic approach. Only patients who agreed to an awake surgical approach and were scheduled for surgery as part of the daily operating room list after initial clinical evaluation were included in the comparative cohort. Among these patients, allocation between WALANT and supraclavicular peripheral nerve block was performed using a planned sequential 1:1 alternating scheme according to the order of enrollment. Patients were allocated into two groups based on the anesthetic method used: the PNB group, consisting of patients who received ultrasound-guided peripheral nerve blocks, and the WALANT group, consisting of patients who underwent surgery using the WALANT technique. To ensure equal group sizes, enrollment continued until 40 patients were included in each group.

### 2.3. Anesthetic Procedures

Sedation was not routinely administered and was used only when clinically required because of patient anxiety, discomfort, or difficulty tolerating the awake operative environment. Standard monitoring, including electrocardiography, non-invasive blood pressure measurement, and peripheral oxygen saturation monitoring, was applied to all patients. Postoperative analgesia consisted of IV paracetamol 1 g administered on demand. No additional rescue analgesic protocol was used.

#### 2.3.1. PNB Group

**Figure 1:**
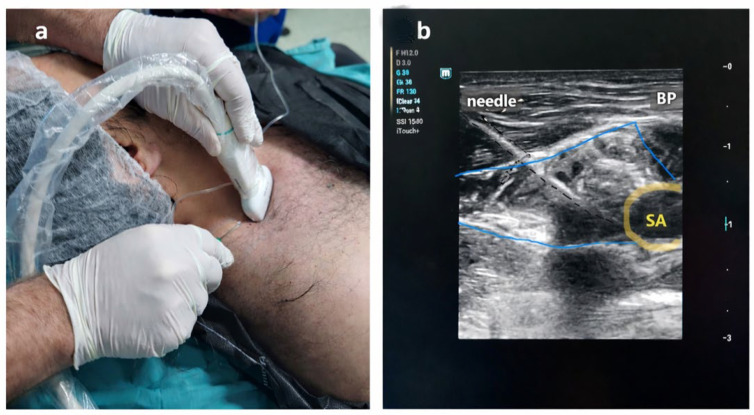
(**a**,**b**). Peripheral nerve block (PNB) application. Performing PNB technique. (**a**) Probe and needle positioning. (**b**) Ultrasound (US) image. The subclavian artery is indicated by a yellow circular marker, and the brachial plexus is outlined with a blue line. The needle trajectory is represented by a black dashed line. PNB, peripheral nerve block; SA, subclavian artery.

In this group, a supraclavicular brachial plexus block was performed under sterile conditions using ultrasound guidance with a linear probe (10–14 MHz). A 22-gauge, 100 mm insulated echogenic needle (Stimuplex^®^ Ultra 360, B. Braun Medical Inc., Melsungen AG, Hessen, Germany) was inserted using an in-plane approach under real-time ultrasound guidance. After careful aspiration, the local anesthetic solution was injected incrementally around the brachial plexus sheath. A mixture consisting of 10 mL of 0.5% bupivacaine, 2.5 mL of 2% lidocaine, and 2.5 mL of normal saline was injected into the perineural area using the hydrodissection technique (total 15 mL). Sensory and motor block success was assessed using the pin-prick test and motor strength examination. Surgery was allowed only after the complete block onset was confirmed. All peripheral nerve blocks were performed by MS. (Figure 1a,b). In the PNB group, a pneumatic tourniquet was routinely applied to provide a bloodless surgical field.

#### 2.3.2. WALANT Group

**Figure 2:**
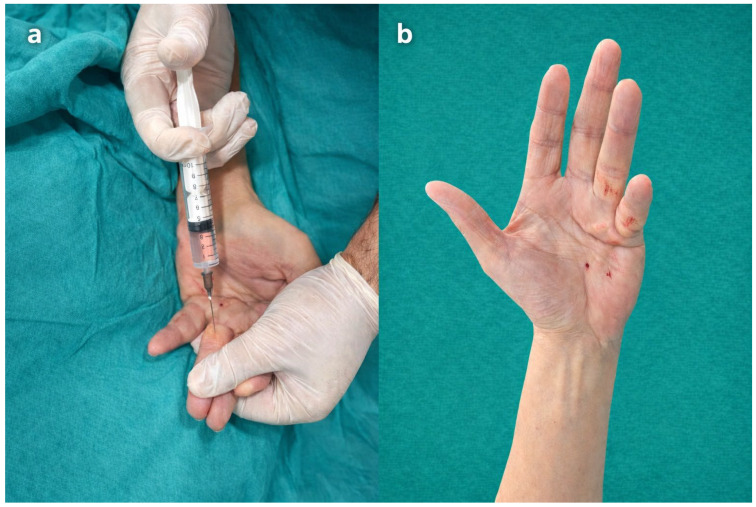
(**a**,**b**). WALANT application. Performing (WALANT) technique. Multiple injections are performed by re-entering the areas affected by the local anesthetic.

In the WALANT group, no tourniquet was applied. A WALANT solution was prepared using 5 mL of 2% lidocaine, 4 mL of normal saline, 1 mL of 8.4% sodium bicarbonate, and 0.1 mL of 1:1000 epinephrine. This mixture corresponded approximately to a final concentration of 1% lidocaine with epinephrine (1:100,000). The solution was infiltrated subcutaneously and subfascially along the surgical incision line and dissection field. The mixture was prepared repeatedly as needed, and a total volume of approximately 25 mL was administered depending on the surgical area. After injection, an appropriate waiting period was observed to allow adequate anesthetic effect and epinephrine-induced vasoconstriction before surgical incision. The total administered lidocaine dose remained within recommended safety limits. All WALANT procedures were performed by surgeons EG and EÖ (Figure 2a,b).

### 2.4. Data Collection and Outcome Measures

All data were recorded in real time using standardized data collection forms prepared by an independent researcher who was not involved in the surgical or anesthetic procedures (MA). All data collection was conducted by two anesthesiologists. (LO and AP). Time intervals were defined as follows:

Block application time (min): Time from the start of anesthetic administration (needle insertion for PNB or initial infiltration for WALANT) to completion of injection. Waiting time (min): Time from completion of anesthetic injection to readiness for surgical incision. Readiness was assessed by the absence of pain to pinprick testing within the planned surgical field in both groups. In the WALANT group, even if adequate anesthesia was achieved earlier, a minimum waiting period of 15 min was observed to allow sufficient epinephrine-induced vasoconstriction before incision. In the PNB group, waiting time corresponded to the time required for sensory block onset confirmed by pinprick testing.

Total procedure time (min): Sum of block application time and waiting time.

Operation time (min): Time from surgical incision to completion of wound dressing.

Pain was assessed using a 0–100 numeric rating scale (NRS), a valid and widely used instrument conceptually similar to a visual analogue scale (VAS), where 0 indicated no pain and 100 indicated the worst imaginable pain. The scale was explained to the patients before assessment and recorded by the study team during the postoperative evaluation. Patients were asked to rate pain during block performance, intraoperative pain, and overall postoperative pain experienced during the first 24 h prior to discharge. Overall patient satisfaction, surgeon satisfaction with the procedure, and willingness to choose the same anesthetic technique again were also assessed using a 0–100 scale. The need for additional intraoperative sedation was recorded as a binary variable. Pain during block performance, intraoperative pain, and surgeon satisfaction were assessed immediately after completion of the procedure, with pain scores reported by patients and surgeon satisfaction recorded by the operating surgeon. Postoperative pain, overall patient satisfaction, and willingness to repeat the same anesthetic technique were assessed on the first postoperative day prior to discharge. Patients were asked to indicate the reasons for satisfaction or dissatisfaction related to the anesthetic technique, with the option to select more than one item. Safety outcomes including technical failure, hematoma, infection, and postoperative motor block were recorded.

### 2.5. Sample Size Calculation

In a previous study, overall patient satisfaction in the WALANT group was reported as 3.27 ± 0.52 on a five-point Likert scale [11]. For planning purposes, we assumed equal spacing between adjacent Likert categories and linearly mapped the 5-point scale to a 0–100 metric to align with the satisfaction scale used in the present study. Under this assumption, the reported standard deviation corresponded to approximately 13 points on a 0–100 scale. A between-group difference of approximately 10 points in overall patient satisfaction was considered clinically meaningful. Based on these assumptions, using a two-sided alpha level of 0.05 and 90% statistical power, the required sample size was estimated to be approximately 36 patients per group. To account for possible exclusions and to ensure balanced group sizes, we planned to include 40 patients in each group.

### 2.6. Statistical Analysis

All statistical analyses were performed using MedCalc Statistical Software, version 23.4.5 (MedCalc Software Ltd., Acacialaan 22, B-8400, Ostend, Belgium). Data distribution was assessed using the Shapiro–Wilk test. As most continuous variables were not normally distributed, they are presented as median [interquartile range], and categorical variables are presented as number (%). Between-group comparisons were performed using the Mann–Whitney U test. Effect size was estimated using the Hodges–Lehmann method with corresponding 95% confidence intervals. Categorical variables were compared using the chi-square or Fisher’s exact test. A two-tailed *p* value < 0.05 was considered statistically significant.

Because this was an observational study, an adjusted analysis was additionally performed for the main primary outcome, overall patient satisfaction, to account for potential confounding. A multivariable regression model was constructed including anesthetic technique as the main independent variable and the following predefined covariates: age, sex, procedure type, operative time, tourniquet use, and additional intraoperative sedation. The adjusted association between anesthetic technique and patient satisfaction was reported with regression coefficients, 95% CIs, and *p* values.

Secondary outcomes were analyzed as exploratory outcomes and were therefore interpreted descriptively without formal adjustment for multiplicity.

Patients requiring conversion to general anesthesia were considered technical failures of the intended awake anesthetic technique and were excluded from the primary comparative outcome analysis according to the predefined study protocol. However, to address potential bias related to post-allocation exclusions, a sensitivity analysis according to the intention-to-treat principle was also performed by retaining converted cases in their originally assigned groups.

Safety outcomes, including conversion to general anesthesia, postoperative motor block, hematoma, infection, and nerve injury, were summarized descriptively for each group.

## 3. Results

A total of 80 patients were included in the analysis, with 40 patients in the PNB group and 40 patients in the WALANT group. All patients completed the postoperative questionnaires and were included in the final analysis. Conversion to general anesthesia occurred in two patients (5%) in the peripheral nerve block group and four patients (10%) in the WALANT group. In the PNB group, conversions were due to block failure. In the WALANT group, two cases were related to inadequate anesthesia and two occurred because of unexpectedly prolonged operative duration. Postoperative motor block was observed in 17 patients (42.5%) in the PNB group. No hematoma or infection was observed in either group (Table 1). The mean age was comparable between groups (32.20 ± 8.68 vs. 34.27 ± 9.79 years, *p* = 0.31). The proportion of female/male patients was similar in the PNB and WALANT groups (13/40 [32.5%] vs. 10/40 [25.0%], *p* = 0.622). Operation time did not differ significantly between groups (72.5 [57.5–90.0] vs. 62.5 [50.0–77.5] min, *p* = 0.112). Demographic characteristics and operative data of the study population are summarized in Table 1.

Block performance time was significantly longer in the PNB group compared with the WALANT group (8 [7–10] vs. 7 [6–8] min, *p* = 0.0023). Waiting time was significantly shorter in the PNB group (15 [12–15.5] vs. 20 [15–20] min, *p* = 0.0005). Consequently, total procedure time was also shorter in the PNB group (23 [20–25] vs. 25 [23–28.5] min, *p* = 0.015) (Table 2).

Overall patient satisfaction was significantly higher in the PNB group compared with the WALANT group (90 [85–100] vs. 80 [70–90], *p* < 0.0001). A sensitivity analysis including the cases converted to general anesthesia according to the intention-to-treat principle yielded results consistent with the primary analysis. After adjustment for age, sex, procedure type, operation time, and intraoperative sedation, the PNB group demonstrated significantly higher overall patient satisfaction scores (adjusted β = 11.88, 95% CI 5.15–18.61; *p* = 0.001). Most patients in both groups reported that they would choose the same anesthetic technique again (38/40 [95%] vs. 35/40 [87%], *p* = NS). Patients’ perceived adequacy of the explanation provided for the procedure was significantly higher in the PNB group compared with the WALANT group (90 [90–100] vs. 85 [80–95], *p* = 0.0006).

Pain during block performance did not differ significantly between groups (20 [10–25] vs. 20 [10–30], *p* = 0.434). Pain experienced during surgery was significantly lower in the PNB group (0 [0–10] vs. 10 [0–20], *p* = 0.026). Postoperative pain scores were also significantly lower in the PNB group (10 [0–20] vs. 30 [5–40], *p* = 0.0048). The requirement for additional intraoperative sedation was similar between groups, occurring in 7 patients (17%) in the PNB group and 8 patients (20%) in the WALANT group (*p* = NS). Postoperative numbness scores did not differ significantly between groups (10 [0–20] vs. 5 [0–20], *p* = 0.188). Surgeon-reported satisfaction scores did not differ significantly between groups (90 [85–100] vs. 90 [80–90], *p* = 0.072).

Analysis of reasons related to patient satisfaction and dissatisfaction demonstrated distinct patterns between the groups. Patients in the PNB group more frequently reported “absence of pain” as a reason for satisfaction [31 (77.5%) vs. 20 (50.0%) *p* = 0.019], whereas patients in the WALANT group more commonly indicated dissatisfaction related to multiple injections [2 (5.0%) vs. 14 (35.0%), *p* = 0.0015] and pain during surgery [4 (10.0%) vs. 12 (30.0%) *p* = 0.048] (Table 3).

## 4. Discussion

The findings of this prospective study suggest that ultrasound-guided peripheral nerve blocks (PNBs) are associated with more effective intraoperative and postoperative analgesia and higher overall patient satisfaction compared with the WALANT technique in selected hand and forearm surgery. In addition, contrary to common perception, PNBs were not associated with prolonged perioperative preparation times and were associated with a shorter time to surgical readiness in the present study.

Patient satisfaction in awake hand and forearm surgery is a multidimensional construct influenced by several perioperative factors. Recent evidence suggests that pain intensity may be associated with patient satisfaction with postoperative care, highlighting the importance of effective pain management across surgical settings [12]. Ultrasound-guided peripheral nerve blocks are associated with improved postoperative analgesia and reduced opioid requirements in hand and forearm surgery, which may support a more favorable early postoperative recovery [13]. However, WALANT is generally applied in selected hand and distal forearm procedures and may not be appropriate for all types of upper extremity surgery. Conflicting findings have been reported in the literature regarding the effect of anesthetic techniques on pain outcomes in hand and forearm surgery. Meunier et al. [6] and Moscato et al. [14], in studies comparing peripheral nerve block and WALANT techniques, reported no statistically significant differences between groups in terms of either intraoperative or postoperative pain scores. Similarly, Sreedevi et al. [15] emphasized the importance of adding sodium bicarbonate to the WALANT solution and using a slow injection technique, reporting lower pain scores during injection compared with the peripheral nerve block group; however, no difference in analgesic efficacy was observed during surgery or in the postoperative period. In contrast, Acar et al. [16] even suggested superior postoperative analgesia in the WALANT group.

In the present study, our findings differ from these reports, as patients in the peripheral nerve block group experienced significantly lower intraoperative and postoperative pain scores. Some patients in the WALANT group reported pain during the procedure as a reason for dissatisfaction. However, intraoperative pain scores measured on the 0–100 scale were generally low, suggesting that these reports likely reflected transient or mild discomfort rather than clinically significant pain. This discrepancy may be partly explained by differences in surgical case mix and the depth of tissue manipulation across studies. In our cohort, the significantly greater postoperative comfort observed in the peripheral nerve block group may be attributed to the longer duration of analgesia provided by long-acting local anesthetics used for nerve blockade, compared with the relatively shorter duration of action of lidocaine-based WALANT solutions. Moreover, pain was assessed separately during block performance, during surgery, and in the postoperative period, allowing a more detailed evaluation of pain experience across different perioperative phases in our study. In addition, postoperative pain assessment covered the entire first 24 h period prior to discharge, thereby encompassing a broader postoperative timeframe compared with studies that rely on single early postoperative measurements. This methodological approach may have facilitated the detection of differences in postoperative analgesic efficacy between anesthetic techniques and may also have influenced the observed difference in patient satisfaction between the two groups. While the lack of tourniquet use in WALANT is generally regarded as a pain-related advantage, the use of a supraclavicular block in our study may have minimized tourniquet-associated discomfort in the peripheral nerve block group, thereby influencing patient satisfaction outcomes [17]. The relatively short operative duration may also have limited the development of tourniquet-related discomfort, particularly in patients receiving peripheral nerve blocks.

Evidence from the perioperative literature indicates that patient satisfaction is influenced not only by analgesic efficacy but also by the quality of communication, patients’ understanding of the procedure, and alignment of expectations, all of which are associated with reduced anxiety and improved overall patient experience [18,19]. Perceived adequacy of explanation may have contributed to overall patient satisfaction in conjunction with pain-related outcomes. Procedural factors, including perioperative workflow and time to surgical readiness, may further influence patient perception. Prolonged waiting periods before incision, even in the absence of pain, can negatively affect the overall experience. In the context of WALANT, the mandatory waiting time required for epinephrine-induced vasoconstriction may partially offset the perceived simplicity of the technique; consistent with this, the total procedural and waiting time was longer in the WALANT group in our study, which may have influenced overall patient satisfaction, particularly in settings where efficient regional anesthesia workflows are available [20]. Taken together, these findings suggest that overall patient satisfaction in our study may have been influenced by a combination of perioperative pain experience, psychological comfort, perceived adequacy of communication, and time-related procedural characteristics.

Consistent with previous reports with high patient acceptability and willingness, our study also demonstrated high rates of repeat preference in both groups, in parallel with high overall patient satisfaction scores [21,22]. From a patient perspective, factors such as avoidance of general anesthesia, rapid recovery, and the ability to remain awake and communicative during surgery may outweigh moderate differences in pain perception when considering future preference. Taken together, these findings suggest that willingness to repeat an anesthetic technique reflects a threshold of acceptability and represents a distinct dimension of patient experience rather than a direct measure of comparative superiority.

Several studies have suggested that WALANT can be applied more rapidly than peripheral nerve blocks and may facilitate earlier transfer of patients to the operating room [23]. In the present study, although block performance time was longer in the PNB group, the total time required to achieve pain-free surgical readiness was significantly shorter compared with the WALANT group. This discrepancy may be explained by differences in block technique selection, as the supraclavicular block is generally associated with a faster onset, as well as by variations in institutional workflow efficiency. Additionally, the prolonged waiting time observed with WALANT in our study is likely related to the mandatory delay required for epinephrine-induced vasoconstriction prior to incision [20].

When the other outcomes of our study were examined, no statistically significant differences were observed between the groups with respect to surgeon satisfaction or the need for additional intraoperative sedation. Verrewaere et al. reported no difference in surgeon satisfaction when comparing locoregional block and WALANT in endoscopic carpal tunnel surgery; however, they did not recommend the WALANT technique in terms of surgical visualization [24]. In contrast, our findings suggest that both techniques appear to provide adequate and safe working conditions for the surgeon with respect to surgical field visibility and hemostasis quality. It should be noted that in peripheral nerve block techniques, the use of diluted local anesthetic solutions may reduce the incidence and intensity of motor blockade, thereby allowing acceptable intraoperative conditions when limited movement is desirable [25]. Moreover, the optimal anesthetic strategy may vary according to the surgical procedure, as intraoperative movement may be required in certain interventions such as tendon repair [26], whereas complete immobility is preferred in others. These procedure-specific requirements should be considered when interpreting surgeon-related outcomes.

From a clinical perspective, these findings suggest that both WALANT and ultrasound-guided peripheral nerve block can be safely used in appropriately selected patients undergoing hand and forearm surgery. However, peripheral nerve block may offer advantages in terms of analgesic efficacy and overall patient satisfaction when regional anesthesia expertise and workflow are available. Conversely, WALANT may remain a valuable option in settings where anesthesiology resources are limited or when surgeon-directed local anesthesia is preferred.

### Limitations

This study has several limitations. First, it was a single-center, prospective observational study in which the anesthetic technique was determined by patient preference rather than randomization; therefore, selection bias and residual confounding cannot be fully excluded. In addition, because cases requiring conversion to general anesthesia were excluded from the comparative analysis, the primary analysis should be interpreted as a per-protocol comparison of procedures successfully completed under the intended anesthetic technique, which may limit generalizability to all initially assigned patients. Second, time-related outcomes may be influenced by institution-specific workflow and team efficiency, which may limit generalizability to settings with different regional anesthesia logistics. Third, the regional anesthesia arm was limited to supraclavicular brachial plexus block, and the findings may not be directly applicable to other brachial plexus approaches (e.g., infraclavicular or axillary block) with different performance and onset profiles. Fourth, outcome assessment was performed by anesthesiologists who were aware of the anesthetic technique, and assessor blinding was therefore not possible. However, the primary outcomes were patient-reported measures, which may reduce the potential influence of assessor-related measurement bias.

## 5. Conclusions

In this prospective comparative study, patient satisfaction was significantly higher in the PNB group than in the WALANT group, and, contrary to the prevailing perception in the literature, this technique was associated with a shorter total anesthesia preparation time. While WALANT achieved acceptable satisfaction levels and provided adequate surgical working conditions, the peripheral nerve block was associated with lower pain scores. These findings suggest that the supraclavicular peripheral nerve block may represent a favorable option when optimizing patient satisfaction is a priority, whereas WALANT remains a safe and feasible alternative in appropriately selected patients.

## Figures and Tables

**Table 1:** Patient demographics and distribution of surgical procedures according to anesthetic technique.

Variable	PNB Group (*n* = 40)	WALANT Group (*n* = 40)	*p* Value
**Age (years),** mean ± SD	32.20 ± 8.68	34.27 ± 9.79	0.31
**Sex, *n* (%)**			
Male	27 (67.5%)	30 (75.0%)	0.622
Female	13 (32.5%)	10 (25.0%)	
**Operation time (min),** median [IQR]	72.5 [57.5–90.0]	62.5 [50.0–77.5]	0.112
**Surgical procedure (*n*%)**			
Hand surgery/Tendon–Nerve– Vessel repair More than one repair Tendon only Nerve only	30 (75%) 21 5 4	29 (72.5%) 19 7 3	0.799
Plate–screw fixation	10 (25.0%)	11 (27.5%)	
**Conversion to general anesthesia (*n*%)**	2 (5%)	4 (10%)	0.400
Technical failure	2	2	
Prolonged operation	0	2	
**Postoperative transient motor block (*n*%)**	17 (42.5%)	0	<0.001
**Hematoma (*n*%)**	0	0	-
**Infection (*n*%)**	0	0	-
**Nerve damage (*n*%)**	0	0	-
**LAST (*n*%)**	0	0	-

Values are presented as *n* (%) and median [interquartile range]. Comparisons between groups were performed using the Mann–Whitney U test for continuous variables and Fisher’s exact test for categorical variables. Conversions to general anesthesia were considered technical failures according to the predefined study protocol and were excluded from the comparative outcome analysis. PNB: Peripheral nerve block, WALANT: Wide Awake Local Anesthesia No Tourniquet, LAST: Local anesthetic systemic toxicity.

**Table 2:** Comparison of perioperative outcomes between WALANT and supraclavicular peripheral nerve block.

Variable	PNB (Group 1) *n* = 40	WALANT (Group 2) *n* = 40	Effect Size/Statistical Test
Block performance time (min)	8 (7–10)	7 (6–8)	HL diff: 2 (95% CI 0 to 2); ***p* = 0.0023**
Waiting time (min)	15 (12–15.5)	20 (15–20)	HL diff: −5 (95% CI −5 to 0); ***p* = 0.0005**
Total procedure time (min)	23 (20–25)	25 (23–28.5)	HL diff: −2 (95% CI −4 to 0); ***p* = 0.015**
Additional sedation, *n* (%)	7 (17%)	8 (20%)	Δ = 3%; 95% CI −14.2 to 20.1; χ^2^ = 0.12; *p* = NS
Postoperative numbness score (0–100)	10 (0–20)	5 (0–20)	HL diff: 0 (95% CI 0 to 10); *p* = 0.188
Pain during block performance (0–100)	20 (10–25)	20 (10–30)	HL diff: 0 (95% CI −10 to 0); *p* = 0.434
Pain during surgery (0–100)	0 (0–10)	10 (0–20)	HL diff: −10 (95% CI −10 to 0); ***p* = 0.026**
Postoperative pain (0–100)	10 (0–20)	30 (5–40)	HL diff: −10 (95% CI −20 to 0); ***p* = 0.0048**
Surgical satisfaction score (0–100)	90 (85–100)	90 (80–90)	HL diff: 0 (95% CI 0 to 10); *p* = 0.072
Adequacy of the explanation for the procedure (0–100)	90 (90–100)	85 (80–95)	HL diff: 10 (95% CI 0 to 10); ***p* = 0.0006**
Overall patient satisfaction (0–100)	90 (85–100)	80 (70–90)	HL diff: 10 (95% CI 10 to 15); ***p* < 0.0001**
Would choose the same technique again, *n* (%)	38/40 (95%)	35/40 (87%)	Δ = 8% (95% CI −5.6 to 22.2); χ^2^/Fisher; *p* = 0.43

Continuous and ordinal outcomes are reported on a 0–100 numeric rating scale and are presented as median (interquartile range) due to non-normal distribution. Between-group comparisons were performed using the Mann–Whitney U test, with effect size estimated by the Hodges–Lehmann method and corresponding 95% confidence intervals. Categorical variables are presented as number (percentage) and were compared using the chi-square test. A two-tailed *p* value < 0.05 was considered statistically significant. Hodges–Lehmann differences are reported as PNB minus WALANT.

**Table 3:** Distribution of Reasons for Patient Satisfaction and Dissatisfaction in the PNB and WALANT Groups.

Reason for Satisfaction	PNB (*n* = 40) *n* (%)	WALANT (*n* = 40) *n* (%)	*p* Value
Being awake during surgery	30 (75.0%)	30 (75.0%)	1.000
Absence of pain	31 (77.5%)	20 (50.0%)	**0.019**
Ability to eat and drink	24 (60.0%)	21 (52.5%)	0.652
Other reasons	2 (5.0%)	2 (5.0%)	1.000
**Reason for dissatisfaction**			
Multiple injections	2 (5.0%)	14 (35.0%)	**0.0015**
Pain during procedure	9 (22.5%)	11 (27.5%)	0.797
Pain during operation	4 (10.0%)	12 (30.0%)	**0.048**

Binary variables were compared between groups using Fisher’s exact test. Each reason for satisfaction or dissatisfaction was analyzed as a dichotomous variable (present/absent). A two-sided *p* value < 0.05 was considered statistically significant.

## Data Availability

Data is available by contacting the corresponding author upon reasonable request.

## Abbreviations

The following abbreviations are used in this manuscript:

WALANT	Wide Awake Local Anesthesia No Tourniquet
PNB	Peripheral Nerve Block
LAST	Local anesthetic systemic toxicity
